# Marek’s Disease Virus Virulence Genes Encode Circular RNAs

**DOI:** 10.1128/jvi.00321-22

**Published:** 2022-04-12

**Authors:** Alexis S. Chasseur, Gabrielle Trozzi, Céline Istasse, Astrid Petit, Perrine Rasschaert, Caroline Denesvre, Benedikt B. Kaufer, Luca D. Bertzbach, Benoît Muylkens, Damien Coupeau

**Affiliations:** a Namur Research Institute for Life Sciences (NARILIS), Integrated Veterinary Research Unit (URVI), University of Namur, Namur, Belgium; b Equipe Transcription et Lymphome Viro-Induit (TLVI), UMR 7261 CNRS, UFR Sciences et Techniques, Université François Rabelais de Tours, Parc de Grandmont, Tours, France; c INRAE, UMR1282 ISP, Equipe Biologie des Virus Aviaires, Nouzilly, France; d Institute of Virology, Freie Universität Berlin, Berlin, Germany; e Veterinary Centre for Resistance Research (TZR), Freie Universität Berlin, Berlin, Germany; University of Arizona

**Keywords:** alternative transcripts, circular RNAs, herpesvirus-induced oncogenesis, Marek’s disease virus

## Abstract

Circular RNAs (circRNAs) are a recently rediscovered class of functional noncoding RNAs that are involved in gene regulation and cancer development. Next-generation sequencing approaches identified circRNA fragments and sequences underlying circularization events in virus-induced cancers. In the present study, we performed viral circRNA expression analysis and full-length sequencing in infections with Marek’s disease virus (MDV), which serves as a model for herpesvirus-induced tumorigenesis. We established inverse PCRs to identify and characterize circRNA expression from the repeat regions of the MDV genome during viral replication, latency, and reactivation. We identified a large variety of viral circRNAs through precise mapping of full-length circular transcripts and detected matching sequences with several viral genes. Hot spots of circRNA expression included the transcriptional unit of the major viral oncogene encoding the Meq protein and the latency-associated transcripts (LATs). Moreover, we performed genome-wide bioinformatic analyses to extract back-splice junctions from lymphoma-derived samples. Using this strategy, we found that circRNAs were abundantly expressed *in vivo* from the same key virulence genes. Strikingly, the observed back-splice junctions do not follow a unique canonical pattern, compatible with the U2-dependent splicing machinery. Numerous noncanonical junctions were observed in viral circRNA sequences characterized from *in vitro* and *in vivo* infections. Given the importance of the genes involved in the transcription of these circRNAs, our study contributes to our understanding and complexity of this deadly pathogen.

**IMPORTANCE** Circular RNAs (circRNAs) were rediscovered in recent years both in physiological and pathological contexts, such as in cancer. Viral circRNAs are encoded by at least two human herpesviruses, the Epstein Barr virus and the Kaposi’s Sarcoma-associated herpesvirus, both associated with the development of lymphoma. Marek’s disease virus (MDV) is a well-established animal model to study virus-induced lymphoma but circRNA expression has not been reported for MDV yet. Our study provided the first evidence of viral circRNAs that were expressed at key steps of the MDV lifecycle using genome-wide analyses of circRNAs. These circRNAs were primarily found in transcriptional units that corresponded to the major MDV virulence factors. In addition, we established a bioinformatics pipeline that offers a new tool to identify circular RNAs in other herpesviruses. This study on the circRNAs provided important insights into major MDV virulence genes and herpesviruses-mediated gene dysregulation.

## INTRODUCTION

Circular RNAs (circRNAs), a new class of noncoding RNAs, were rediscovered in recent years because of increased interest in the regulation of gene expression by noncoding RNAs (ncRNAs). As their name indicates, circRNAs are different from linear RNAs as they are continuous covalently closed loops without a 5′-cap structure and a 3′-polyA tail. They result from back-splicing events, defined by the head-to-tail “back-splicing” of a downstream splice donor to a more upstream splice acceptor. Most of the characterized circularizations have been linked to the canonical splicing machinery that is dependent on the U2 spliceosome to date (1, 2). However, several examples of circRNAs for which the nucleotide signature at back-splicing sites does not match the U2 processing pattern have been described in different animals and plants (3–7). These are considered noncanonical back-splicing sites. Due to their circular structure that is naturally resistant to degradation by exonucleases, circRNAs are highly stable and some of them are more abundant compared to their linear counterparts (8). CircRNAs are conserved through evolution and are often specifically expressed according to developmental stages and tissue origins. CircRNAs can act (i) by sponging the effect of microRNAs (miRNAs) (9, 10); (ii) by regulating gene expression at the transcriptional and posttranscriptional levels through various mechanisms (11, 12); (iii) and eventually as a coding template through a cap-independent translation mechanism (13). Aberrant circRNA expression has been identified as a hallmark of cancer (14–16).

The pattern of host circRNA expression is modified during numerous viral infections (17–20). However, only a few viruses have been shown to encode their circRNAs. Most of the viral circRNAs have been identified in the *Herpesviridae* family (6, 21, 22) but some circRNAs are produced by other DNA virus families, such as the *Papillomaviridae* (23) and *Polyomaviridae* (24) or RNA viruses, e.g., *Coronaviridae* (25) or *Paramyxoviridae* (26). Former functional studies on herpesvirus-encoded circRNAs have suggested roles in immune evasion and cancer development (27–29).

Marek's disease is a neoplastic disease of chickens causing severe economic hardships to the poultry industry (30). In addition to its negative economic impact, Marek’s disease virus (MDV) is frequently used as a well-established model to study the different steps of virus-induced lymphoma development in its natural host, as well as the role of ncRNAs in this process (31–33). This avian alphaherpesvirus shares several properties with human gammaherpesviruses, such as Epstein Barr virus (EBV) and Kaposi’s Sarcoma-associated herpesvirus (KSHV), which are associated with tumor development under specific conditions in latently infected lymphoid cells (reviewed in reference (34)). MDV regulates viral and cellular genes through diverse mechanisms during the four stages of viral infection: the early production phase, the latent infection phase, and the late productive phase that is followed by reactivation, and cellular transformation. The major MDV oncoprotein Meq plays an essential role in tumorigenesis (35–38). In addition, the role of numerous linear noncoding RNAs, including the viral microRNAs (39, 40), the viral telomerase RNA subunit (vTR) (41–43), and two viral long noncoding RNAs (lncRNAs) have been investigated. These two lncRNAs are encoded by various splicing isoforms from the repeat regions surrounding the unique long regions (IR_L_/TR_L_) for the edited repeat long (ERL) (44) and the unique short regions (IR_S_/TR_S_) for the latency-associated transcripts (LATs) (39, 45, 46).

Because circRNAs have been identified as key regulators in tumorigenesis and more specifically in virus-induced cancers, we set to decipher their expression in MDV infection and tumorigenesis. In this study, we identified viral circRNA expression at key steps of the MDV infection cycle. Complementary approaches demonstrated that major MDV virulence genes express a huge range of circRNAs. Thorough analyses of circularization sites highlighted the use of both the canonical and noncanonical back-splicing machinery. Moreover, analyses of MDV-induced tumors revealed peaks of viral circRNA expression from latency- and cancer-related genes. Consequently, this study broadens the scope of noncoding RNA functions in Marek’s disease development.

## RESULTS

### Identification of MDV loci-expressing circRNAs.

While previous studies have reported on the expression of host circRNAs during MDV infection (20, 47), no study has addressed the expression of MDV-encoded circRNAs until now. Thus, we investigated MDV-encoded circRNAs from two transcriptional units involved in viral pathogenesis and tumorigenesis, namely, the *meq* oncogene and the LATs. These two genes are localized in the repeat regions of the genome from which abundant and linear alternative transcripts derived from complex splicing were discovered (39, 44, 48–50). An RT-PCR approach, based on divergent primers that exclusively amplify circRNAs, was applied to total RNA extracts depleted of linear transcripts. This method was carried out on several relevant biological samples associated with the different phases of the viral cycle: (i) two commonly used permissive cell types (chicken embryonic fibroblasts, CEF, and ESCDL-1 cells) infected with the very virulent RB-1B strain as a model for the productive phases of the virus; (ii) a lymphoma-derived cell line (MSB1) as a model for transformed T cells infected with MDV; (iii) MSB1 cells treated with sodium butyrate (NaBu) or 5’azacytidine as a model for virus reactivation as demonstrated previously (51); (iv) three samples harvested from RB-1B infected chickens (peripheral blood lymphocytes, spleen, and feather follicle epithelium) *ex vivo* RB-1B-infected T and B lymphocytes (52).

**FIG 1:**
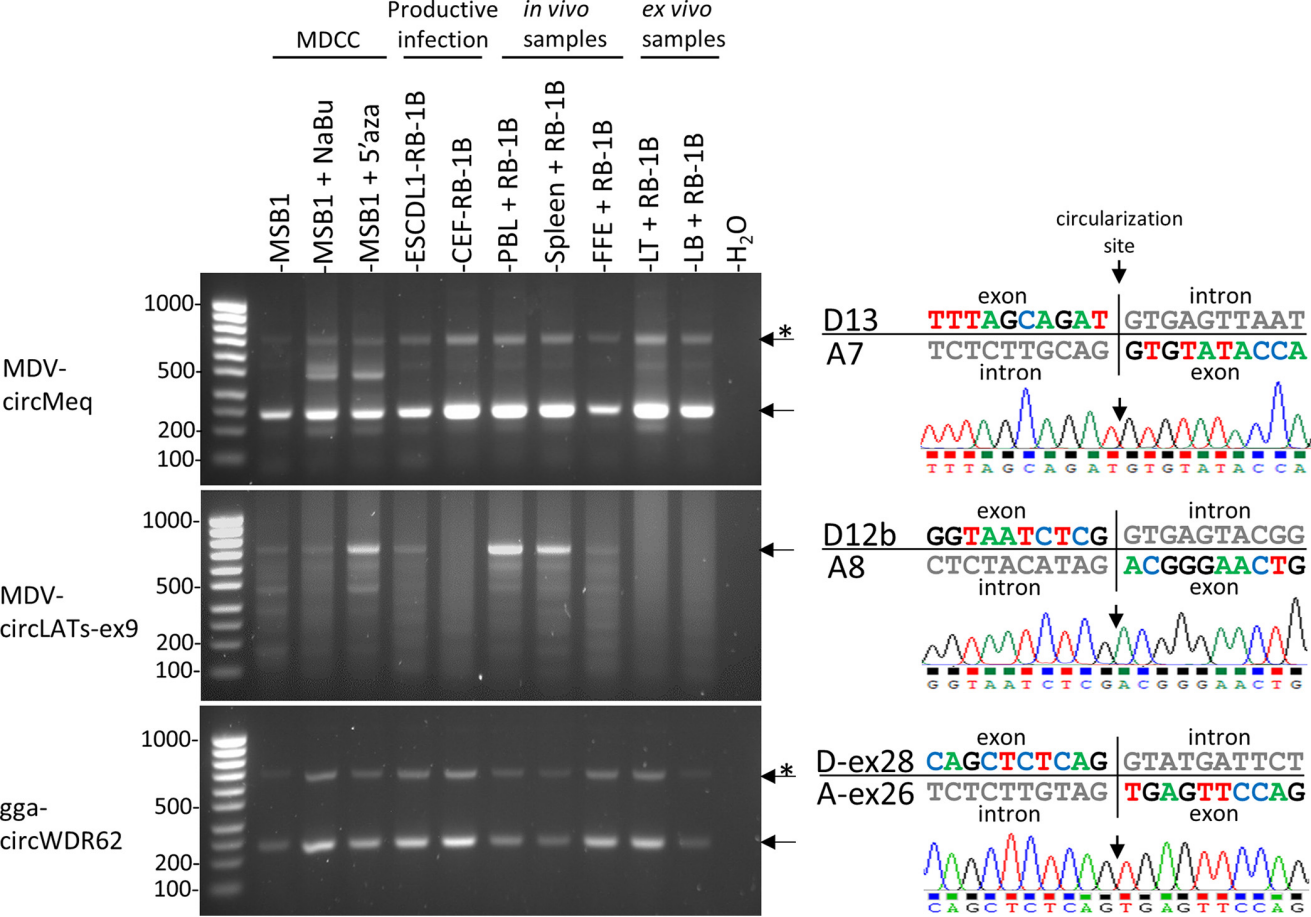
Expression profiles of viral and cellular circRNAs in MDV infected cells that represent the different stages of viral infection. Agarose gel electrophoresis of inverse RT-PCR products obtained from two viral (*meq* and *LATs*) and one cellular (*WDR62*) gene are presented on the left part. Corresponding chromatogram results of the sequenced back-junctions obtained from the major amplicons are presented on the right. Both the exonic and intronic sequences of the donor and acceptor splice sites are indicated. MDCC: Marek’s disease chicken cell; FFE (Feather follicle epithelia); LT: *ex vivo* infected T lymphocytes; LB: *ex vivo* infected B lymphocytes; Asterisks (*) indicate rolling circle amplicons. Ladder: SmartLadder SF 100 bp-1kb (Eurogentec).

Inverse PCR products corresponding to circRNAs were observed from the two investigated loci in various conditions of the viral infection (Fig. 1). Several amplicons were obtained for both loci, which is indicative of circRNA isoforms processed from the *meq* and LATs transcriptional units. While the *meq* locus showed a constitutive expression of circRNAs regardless of the tested RNA sample, circRNA expression from the LATs locus varied according to the source of the RNA. PCR signals specific for circular transcripts were more obvious from PBL and splenocytes collected from *in vivo* infected animals and from lymphoma-derived cells reactivated with 5’azacytidine (Fig. 1). Sanger sequencing of the inverse PCR-amplified *meq* and LATs products confirms the circularization event with the use of an acceptor splice site localized downstream of the donor splice site (Fig. 1). This discovery of the back-splice sites provides the first solid evidence that MDV encodes circular RNAs *in vitro* and *in vivo*.

**FIG 2:**
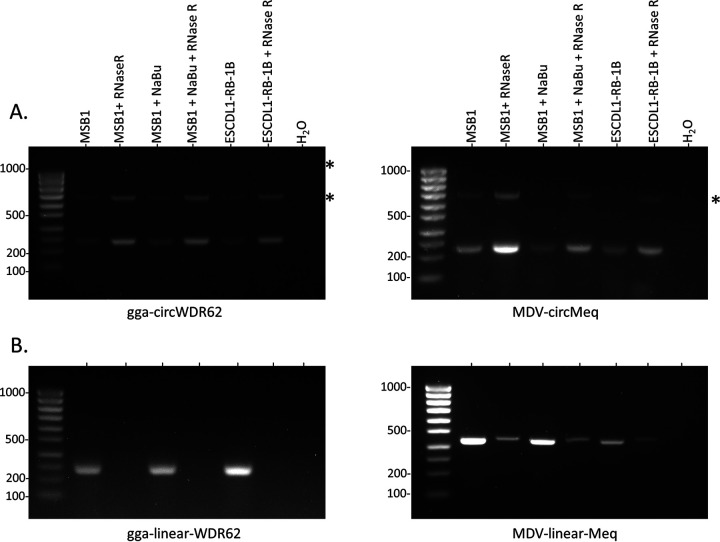
Effect of RNase R treatment on circular RNA enrichment. Agarose gel electrophoresis of RT-PCR products of circular (A) and linear (B) gga-*WDR62* and MDV-*meq* was obtained from three samples untreated or treated with RNase R before the reverse transcription procedure as indicated. Ladder: SmartLadder SF 100 bp to 1kb (Eurogentec). Asterisks (*) indicate rolling circle amplicons.

The robustness of this method, based on inverse PCR amplification to identify circRNAs, was validated on both viral and cellular genes by assessing the enrichment of circular transcripts and degradation of their linear counterparts after RNase R treatment (Fig. 2). We performed PCR assays to monitor circular and linear forms of viral *meq* and host gga-WDR62 transcripts (53). The signal increase observed in Fig. 2A ensured the circular shape of both transcripts. The signal decrease seen in Fig. 2B showed that RNase R treatment targets and degrades linear transcripts.

### Identification of canonical circRNAs expressed in the MDV repeat regions.

After identifying the expression of circRNAs from two key virulence genes of the virus, we explored the transcription of circRNAs from the repeat regions of the MDV genome, which is known to express most of the pathogenesis-related genes as multiple spliced transcripts, in a nonexhaustive way. Twenty-eight primer pairs (19 in TR_L_/IR_L_ and 9 in IR_S_/TR_S_) were designed to target previously identified exons of pp14, *meq*/vIL-8, ERL, or LATs loci (Table 1). We used two cell lines to assess the different stages of the MDV infection cycle: ESCDL-1 cells infected with the MDV RB-1B strain representing lytic viral replication; MSB1 as a representative cell line of latency and transformation; and MSB1 cells treated with NaBu to mimic virus reactivation.

**TABLE 1:** Primers used in this study^*a*^

No. PCR	PCR target	Orientation	Sequence (5′–3′)	Position on TRL or IRS	Position on IRL or TRS
1	pp14-ex1	Fwd	CGATCCCCGATATATTTCCT	13791-13772	129209-129228
		Rev	CGTGCCATTCTGAGAGAGCA	13794-13813	129206-129187
2	pp14-ex1	Fwd	GAGCTGAATTTCTCCCTTCATC	13743-13722	129257-129278
		Rev	CGTGCCATTCTGAGAGAGCA	13794-13813	129206-129187
3	pp14-ex2	Fwd	GTCGATTGACACGGCTCTG	10867-10849	131473-131491
		Rev	GACGTTGATGGAGGAGTTGC	10927-10946	131413-131394
4	ERL-ex9	Fwd	GCTTCCGTCTTTGTACATGATAA	10921-10899	131419-131441
		Rev	GACGTTGATGGAGGAGTTGC	10927-10946	131413-131394
5	pp14-ex3	Fwd	CAACTAGGAACGGAACATCAAGT	9748-9726	132592-132614
		Rev	AGTGGGTCCGCAGTCAATGC	9774-9793	132566-132547
6	pp14-ex3	Fwd	GGTTCTGGCAGAGATTCCAC	9793-9774	132704-132723
		Rev	AGTGGGTCCGCAGTCAATGC	9774-9793	132566-132547
7	ERL-ex8	Fwd	CCATCTCGAAACGGATACTGC	9771-9751	132569-132589
		Rev	GGTCTGCAACGATATCACAGC	9800-9820	132540-132520
8	ERL-ex7	Fwd	AGATGTTGTAGGGTTCGAGAGG	9180-9159	133159-133180
		Rev	ACGACCGACAGAGAAAGTTGG	9249-9269	133090-133070
9	RLORF6/ERL-ex6	Fwd	AACGATTGCGGAAGTACGGC	8686-8667	133653-133672
		Rev	CTGCAAACTATATCGGAATGCG	8853-8874	133486-133465
10	ERL-ex5	Fwd	TCGTGACATTTAGAACCAAAGTG	7542-7520	134797-134819
		Rev	AGCCTTAAGTAGAATAACTGGCG	7599-7621	134740-134718
11	miRNA cluster M9M4	Fwd	CTGGGGGAAATGATCGCTGG	6266-6247	136074-136093
		Rev	ATACGCGGTGTCACGAAGACCGA	6518-6540	135822-135800
12	Meq	Fwd	CACCCCTTCCCTGACGGCCTATC	5690-5668	136652-136674
		Rev	GTGCCCGCCTTCTCCCTGGTATAC	5843-5866	136499-136476
13	ERL-ex3	Fwd	TATGCCCTACAGTCCCGCTG	5799-5780	136543-136562
		Rev	GAACGGTCACAATTCACCTGTC	6005-6026	136337-136316
14	ERL-ex2	Fwd	TCAGCAGCACATCGTCTATGC	4787-4767	137555-137575
		Rev	CCGAATACAAGGAATCCTGTTC	4880-4901	137462-137441
15	RLORF5a/ERL-ex1	Fwd	TATGCTTTCTCTACGATGTGGCA	3673-3651	138668-138690
		Rev	CGCAGATTACTCCTGCATAAGC	3857-3878	138484-138463
16	RLOF5a	Fwd	AGAGCATGAAAATTAAATCGTAGC	3419-3396	138922-138945
		Rev	CCCTTCCCGTTCACTCTTTC	3487-3506	138854-138835
17	RLORF4	Fwd	AGACCCAATAACAGGGAAATCG	2711-2690	139629-139650
		Rev	GGGCATAAACTATAGCATCGAA	2798-2819	139542-139521
18	vIL-8-ex2	Fwd	CTGTTGACGTGATACCACCG	2285-2266	140055-140074
		Rev	ACAGCGAGACTCTCCAGTGATA	2348-2369	139992-139971
19	vIL-8-ex3	Fwd	ACACAATTGAGCCCACACCTC	1983-1963	140357-140377
		Rev	CACATACCTTCCTGTTCTTCTTGAG	2107-2131	140233-140209
20	LAT ex1	Fwd	CCGCAGGTCTCTCGGACTAG	142813-142832	177550-177531
		Rev	CGCCAAACTTGTGCCAAAC	142773-142755	177590-177608
21	LAT ex2	Fwd	TTCTTTTGCGTCTGCCGAC	144509-144527	175854-175836
		Rev	TACGATCTATCGGACGAACCAC	144382-144361	175981-176002
22	LAT ex3	Fwd	CGGGAATGTGAACAACAACC	145206-145225	175157-175138
		Rev	GAAGATATGGTAGCCTCAATTCG	145182-145160	175181-175203
23	LAT ex5	Fwd	TCGGGTGCTGCTTTCCATC	145877-145895	174486-174468
		Rev	TATCTCCACTGACTCGGATG	145874-145855	174489-174508
24	LAT ex7	Fwd	GTTTGACCGATCACCGTTCC	146342-146361	174021-174002
		Rev	CCGAGGCTATGGCAGACATC	146234-146215	174129-174148
25	LAT ex9	Fwd	ATGAAGAGGATGGCGATCTGG	148764-148784	171599-171579
		Rev	CGTAAACTGGAAGATGAGGAC	148566-148546	171797-171817
26	LAT ex11	Fwd	TCTCAAAAGTAACTTCGCAGCC	149418-149439	171945-171924
		Rev	CCACAACCATCTACCACGCTG	149415-149395	170948-170968
27	LAT ex13	Fwd	CCACAATTGCAAAGTGCGGTA	152320-152340	168043-168023
		Rev	TCGGGCAGCAATCAGATACG	152277-152258	168086-168105
28	LAT ex15	Fwd	TCGGGGTTCTGTACGATTGG	153647-153666	166716-166697
		Rev	TTTTGTTTACTTGCAGATATGCACC	153645-153621	166718-166742
	gga-circWDR62	Fwd	CTCAAGCAGCATTTTGGGACC	NA	NA
		Rev	GTCTGGGCTGGAAAGACGAC	NA	NA
	gga-linear WDR62	Fwd	CTCCTACATCTGTAACTGCCTTGG	NA	NA
		Rev	GTATCTTGTGGCTTCGTCAGTACC	NA	NA

a Primers were designed according to the sequence of the MDV RB-1B genome.

**FIG 3:**
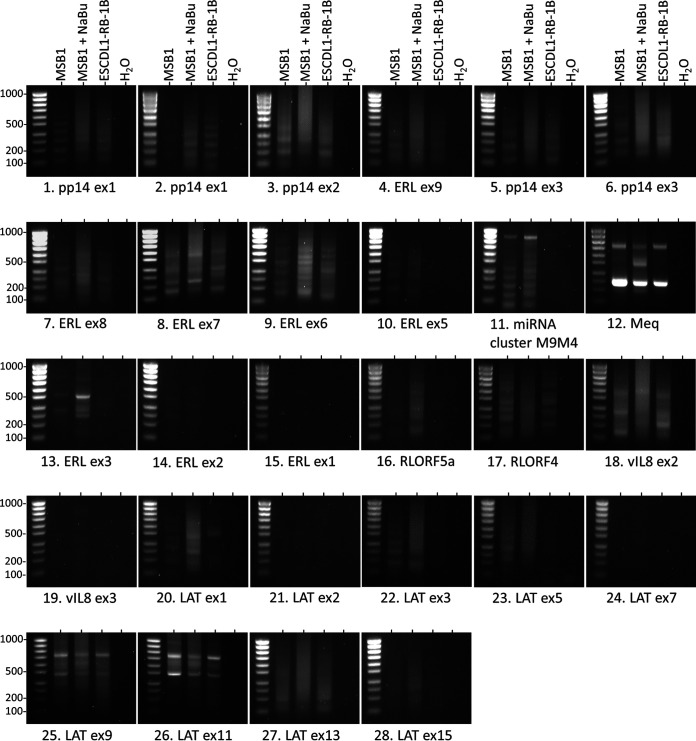
Agarose gel electrophoresis of inverse RT-PCR products obtained for the 28 investigated viral loci. The three tested cDNAs are representative of the three stages of the viral infection, MSB1: latency, MSB1 + NaBu: reactivation; ESCDL-1-RB-1B: lytic replication. Ladder: SmartLadder SF 100 bp to 1kb (Eurogentec).

By using the inverted PCR approach, an abundant and diversified circRNA expression profile was determined in most of the tested loci (Fig. 3). Although we observed differential expression for several amplicons according to the investigated stages of the viral infection, the strategy aimed at describing the circRNAome in a nonquantitative way. Each positive RT-PCR product was then purified, cloned, and sequenced, leading to the identification of 518 sequences containing back-splicing events. Among these sequences, 156 were processed through U2 canonical back-splicing. After alignment, three loci were highlighted that encode all these canonical circRNAs. We identified nine canonical circRNAs from the miRNAs/*meq*/vIL-8 locus, 10 from the ERL lncRNA (Fig. 3) and 28 from the LAT region between exon 1 to 12 (Fig. 3).

**FIG 4:**
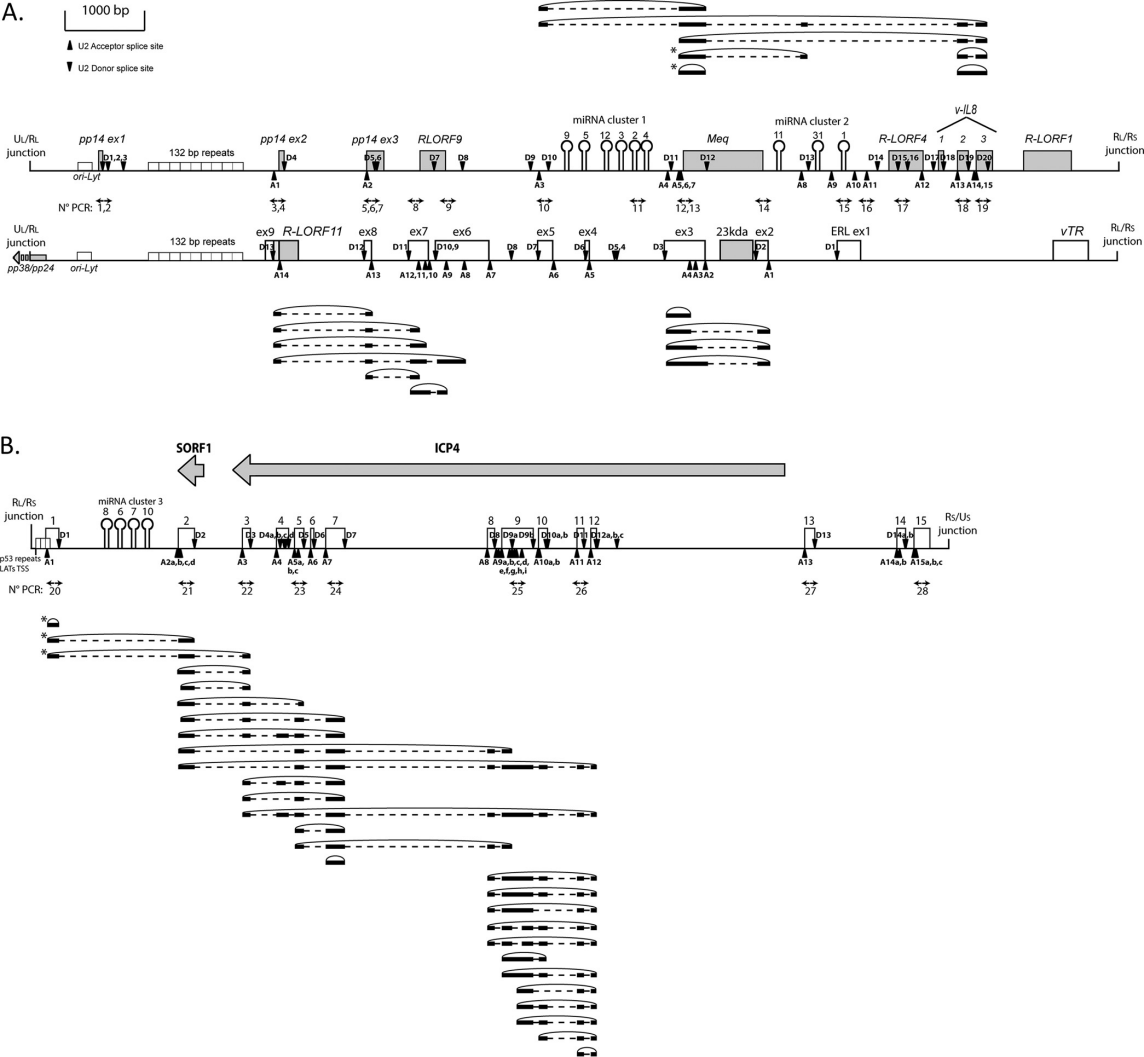
Canonical circRNAs expressed by the MDV repeat regions. (A) Localizations of the circRNAs were processed through the canonical U2 machinery and identified in the MDV IR_L_. Both strands of the viral genome (RB-1B) from nucleotide 128,000 to nucleotide 142,000 and their associated ORFs are represented in the center. (B) Localizations of the circRNAs were processed through canonical U2 machinery and identified in the MDV IR_S_. Both strands of the viral genome from nucleotide 142,500 to nucleotide 154,000 and their associated ORFs are represented at the top. The circRNAs derived from the positive-strand are schematized at the top and circRNAs derived from the negative-strand are schematized at the bottom. Gray boxes represent validated ORFs, and white boxes represent ncRNAs. MiRNAs are represented by hairpins and genomic repetitions by small white boxes. Black triangles and inverted black triangles represent the U2 acceptor splice site (A) and U2 donor splice site (D), respectively. Black lines represent the exonic part and black dotted lines represent the intronic part of the circRNAs. The position of divergent primers and the associated PCR numbers are represented by double arrows between the two schematized genomic strands. Asterisks (*) indicate the use of alternative splice sites.

Interestingly, circRNAs arose from both strands in the TR_L_/IR_L_ regions of the MDV genome. While the nine circular transcripts we found in the *meq*/vIL-8 are in the same orientation as the related Open reading frames (ORFs), the 10 ERL circRNAs were produced from the opposite strand being antisense to parts of pp14 and *meq* ORFs (44) (Fig. 4). These circRNAs are mainly constituted with previously described exons found in linear transcripts (39, 44, 48–50), but we identified new donor or acceptor splice sites that lead to the RNA circularization in the first or the last exons of the linear transcripts like the third exon of vIL-8 or the first exon of the LATs (Fig. 4). The majority of these circRNAs contain multiple exons but a few circRNAs in the explored loci are constituted by only one exon. All the donor and acceptor splice sites are listed with their back-splicing association in Table S1. Altogether, viral circRNAs identified in the tested conditions present a high level of internal alternative splicings, such as intron retention, the use of an alternative donor or acceptor splice site, and exon skipping.

### Identification of noncanonical circRNAs expressed in the MDV repeat regions.

**FIG 5:**
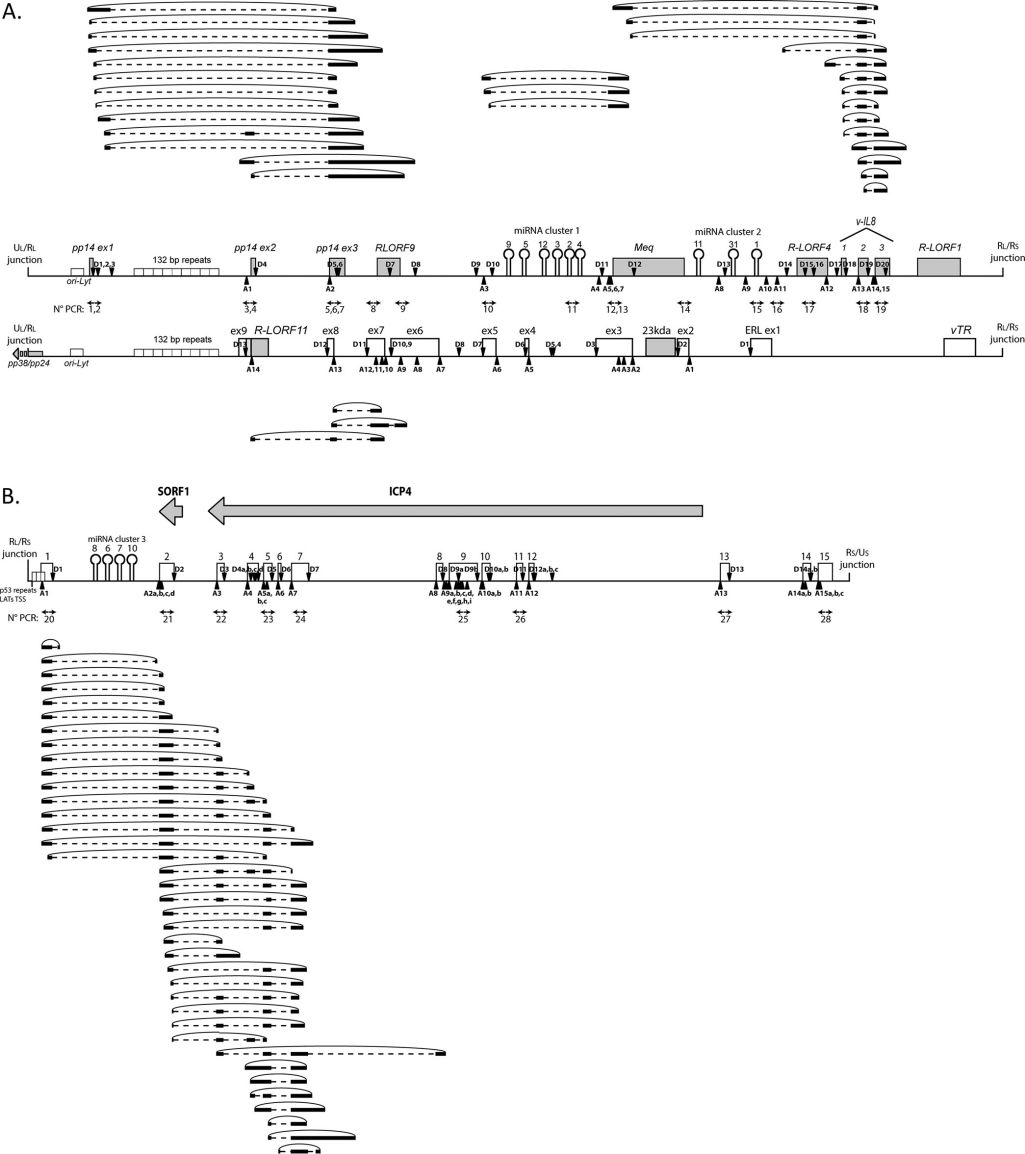
Noncanonical circRNAs expressed by the MDV repeat regions. (A) Localizations of the circRNAs processed through the noncanonical machinery and identified in the MDV IR_L_. Both strands of the viral genome (RB-1B) from nucleotide 128,000 to nucleotide 142,000 and their associated ORFs are represented in the center. (B) Localizations of the circRNAs processed through the noncanonical machinery and identified in the MDV IR_S_. Both strands of the viral genome from nucleotide 142,500 to nucleotide 154,000 and their associated ORFs are represented at the top. The circRNAs derived from the positive-strand are schematized at the top and circRNAs derived from the negative-strand are schematized at the bottom. Gray boxes represent validated ORFs, and white boxes represent ncRNAs. MiRNAs are represented by hairpins and genomic repetitions by small white boxes. Black triangles and inverted black triangles represent the U2 acceptor splice site (A) and U2 donor splice site (D), respectively. Black lines represent the exonic part and black dotted lines represent the intronic part of the circRNAs. The position of divergent primers and the associated PCR numbers are represented by double arrows between the two schematized genomic strands.

Besides identifying circRNAs with canonical U2 back-splicing junction, the thorough analyses carried out on oriented transcripts revealed numerous circular sequences produced from noncanonical back-splicing events. To define the polarity of the matrix strand, the analyses were limited to transcripts encompassing at least one internal canonical splicing event. By using this selection, we identified 70 noncanonical circRNAs showing a complex alternative splicing pattern: 33 spliced noncanonical circRNAs in the R_L_ (Fig. 5A) and 37 in the R_S_ (Fig. 5B). Back-splicing events leading to the generation of these noncanonical circRNAs are localized in intronic or exonic parts of the splice sites.

For the TR_L_/IR_L_, we detected 13 noncanonical circRNAs in the sense strand of the pp14 locus (Fig. 5A). These circRNAs encompass the three pp14 exons and RLORF9 and follow the internal splicing identified for linear transcripts (50). For the miRNAs/*meq*/vIL-8 locus, 17 noncanonical circRNAs were identified (Fig. 5A). These circRNAs encompassed the miRNAs M9-M4 cluster and *meq*/vIL-8 transcript and as observed for the linear spliced transcripts, miRNAs are localized in the intronic part (48). Interestingly, the majority of these circRNAs use a donor back-splice site in the third exon of vIL-8. For the ERL locus, we found three noncanonical circRNAs in the 3′ end of the ERL transcripts (Fig. 5A). As stated above for canonical ERL-derived circRNAs, these noncanonical circRNAs are antisense to pp14. In the IR_S_/TR_S_, the whole series of 37 noncanonical circRNAs derived from the 5′ part of the LATs (from exons 1 to 8), while only canonical circRNAs covered exons 8 to 12 (Fig. 5B). In addition, among the 518 candidate MDV circRNA clones, we analyzed 289 sequences devoid of internal splicing events and processed by noncanonical back-splicing. These circRNAs possess a high variability in size, ranging from 50 to 1428 nt, and in localization (unpublished data).

### Identification of viral circRNAs during *in vivo* infection.

To demonstrate MDV circRNA expression during tumorigenesis in the natural host, we used a complementary approach because our inverse PCR approach was limited to targeted regions. We scanned the full-length viral genome to detect circRNA expression by taking advantage of high-throughput RNAseq data obtained after MDV *in vivo* infections. A bioinformatic pipeline was applied to extract viral sequences from a circRNA enriched data set (accession number GSE138600) that was previously exploited to characterize specific host circRNA signatures during Marek’s disease virus infection (20). This pipeline can identify and quantify new back-spliced junctions in viral transcripts at a nucleotide-precision level, even the noncanonical ones. This analysis was conducted on nine infected animals among which four showed tumor development in the spleen (tumor-bearing animals, TA^+^) and five that did not develop tumors (tumor-exempt animals, TA^−^) (20).

**FIG 6:**
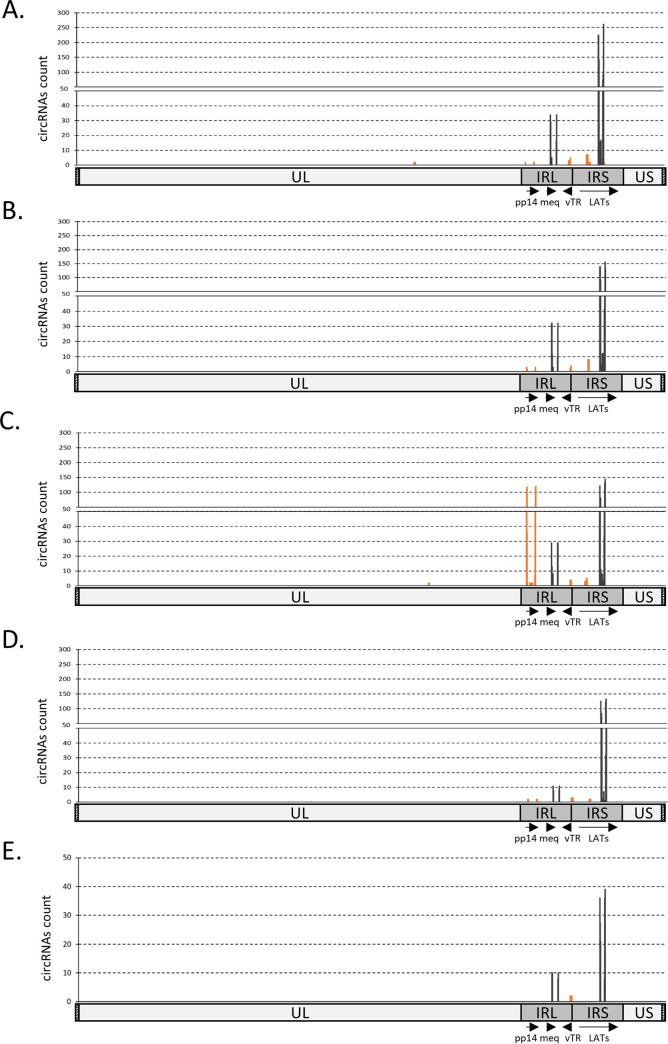
*In vivo* expression of MDV circular RNAs. Localization plots of identified circRNAs from five *in vivo* RNA-seq samples (A to E), mapped on the full-length MDV genome (without the terminal repeat regions) represented on the *x*-axis. The peaks represent raw counts of the mapped reads. The reads were filtered on the fact that they encompass a back-splicing event. Each plot (A to D) represents the mapping of circRNAs extracted from a single infected animal with MDV-induced tumors (TA^+^). (E) The mapping of circRNAs extracted from a single infected animal that did survive the viral infection (TA^−^). Dark lines represent the coverage of identified canonical U2 circRNAs and orange lines represent the coverage of identified noncanonical circRNAs.

After data processing and analyses, we identified numerous circRNAs expressed during MDV pathogenesis. Back-spliced junction counting and localization over the MDV genome revealed a common circRNA expression pattern for the four TA^+^ samples (Fig. 6A to D). In contrast, data analysis from TA^−^ samples did not lead to any circRNA mapping over MDV except for one sample (Fig. 6E). This sample presented the same profile as the TA^+^ with a 5-fold lower circRNA hit count. We found four major loci with viral circRNA expression in the repeat regions, half of them corresponding to U2 back-splicing products and the other half to noncanonical ones. Strikingly the hit counts of the two U2-processed loci comprise the majority of the viral circRNAs. They were identified by open, unbiased sequence analysis and corresponded to the circRNAs identified by the inverse PCR strategy targeting key virulence genes, the LATs, and *meq* transcriptional units (Fig. 1). Back-spliced junction sites, spreading over the LATs exons 8 to 12, constituted 48% to 87% of circRNA hits in TA^+^ samples while those spreading over the *meq* exons were 7% to 15% (Fig. 6).

In addition to U2 circRNAs, several noncanonical circRNAs were mapped in two main loci corresponding to pp14 and vTR transcriptional units (Fig. 6). TA^+^ sample analyses revealed circRNAs processed from noncanonical back-splicing at the pp14 locus, with a differential rate according to the tested samples. Three of the TA^+^ samples presented a low number of pp14 circRNAs while one TA^+^ sample harbored a high noncanonical circRNA expression from this gene corresponding to 39% of all viral circRNAs (Fig. 6C). In contrast, we found noncanonical back-splicing sites at the vTR locus at low levels but consistently in the different spleen samples (Fig. 6).

**FIG 7:**
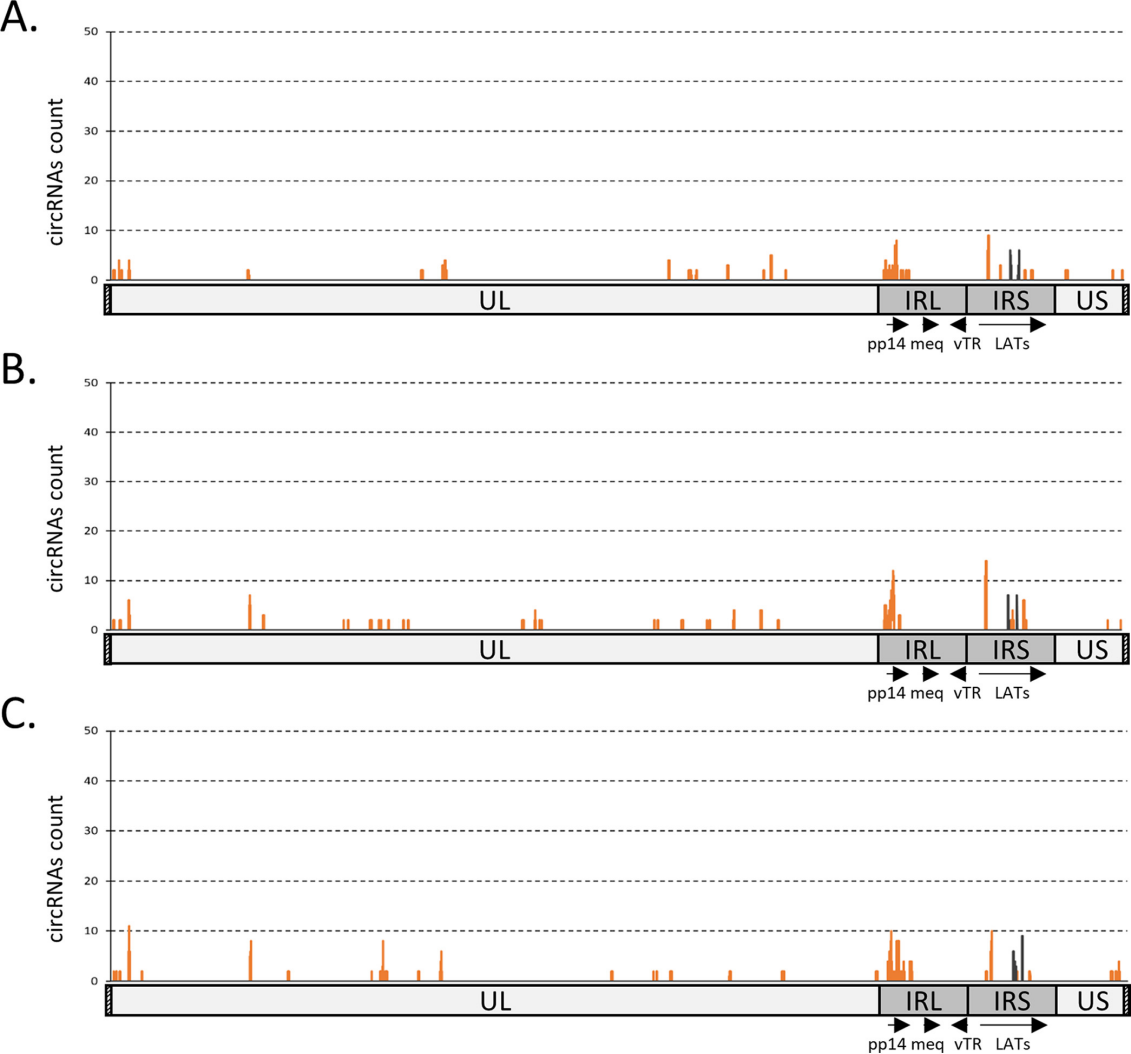
Deep sequencing analysis of MDV circRNAs expression. Localization plots of identified circRNAs from three *in vitro* RNA-seq samples, mapped on the full-length MDV genome (without the terminal repeat regions) represented on the *x*-axis. The peaks represent raw counts of the mapped reads. The reads were filtered on the fact that they encompass a back-splicing event. Each plot represents the mapping of circRNAs extracted from three independent infected CEF cultures (A to C). Dark lines represent the coverage of identified canonical U2 circRNAs and orange lines represent the coverage of identified noncanonical circRNAs.

The bioinformatic pipeline was also applied to another set of high-throughput RNAseq data obtained from an independent experiment investigating MDV *in vitro* productive infection and focusing on host circRNAs dysregulation (47). This RNAseq data set (accession number GSE166240) from infected chicken embryo fibroblasts (CEF) was processed to map back-spliced junctions over the MDV genome. This analysis showed a wider distribution of back-splicing sites and a higher proportion of noncanonical circRNAs during lytic MDV replication (Fig. 7). Canonical circRNAs were only identified from the LATs exon 8 to 12 locus. The highest density and variety of circRNAs are derived from the pp14 locus.

## DISCUSSION

We designed this study to investigate whether an oncogenic avian herpesvirus expresses circRNAs at key steps of viral infection. Our strategy first focused on virulence genes with previously identified complex splicing patterns. The approach was then broadened to identify viral circRNA expression *in vivo* from any MDV transcriptional unit by using a newly developed bioinformatic pipeline that we set up to identify circRNA junction sites from existing RNA sequencing data.

The first series of experiments identified four hot spots of circRNA expression in virulence genes located in the repeat regions of the MDV genome. For some of the target transcripts, viral circRNA expression varied according to the investigated stage of the viral infection, lytic replication, latency, or reactivation. In-depth sequence analyses of each back-splicing site revealed the production of canonical and noncanonical viral circRNAs processed by the U2-dependent or U2-independent splicing machinery, respectively. Therefore, this report provides new information on the production of noncanonical circRNAs during viral infection (6, 25, 26). The next set of experiments was based on open and unbiased analyses of lymphoma-derived sequences over the whole MDV genome and pointed out the *meq* and LATs loci as the central hubs of viral circRNA expression during tumorigenesis. This complementary and independent approach confirmed the production of noncanonical circRNAs through the description of back-spliced junctions, at the nucleotide precision level. Altogether, our data provided the first characterization of circRNA expression in Marek’s disease virus infections. MDV circRNA expression is infection stage-dependent and circRNAs are processed from virulence- and latency-associated transcripts.

The identification of circRNAs encoded from previously characterized genes that play key roles in Marek’s disease pathogenesis challenges our current knowledge and assigned functions of these virulence factors. Indeed, studies addressing the roles of *meq* (35–38, 54, 55), pp14 (50, 56, 57), LATs (39, 45, 46), and ERL (44), ignored the existence of these original circular transcripts. In that regard, identification of circRNA expression during KSHV and EBV infection from previously characterized loci extended the functions of the related genes. In EBV, three gene loci (BART, LMP2, and BHLF1) have been shown to encode circRNAs (21, 58, 59). Among them, functional characterization revealed that circBART2.2 promotes immune escape by regulating programmed cell death-ligand 1 (27) while circ-LMP2a induces stemness in EBV-associated gastric cancers through hsa-miR-3908 sponging (28). In KSHV, different studies identified viral circRNA expression (6, 60, 61). The KSHV circRNAs have been localized in ORFs of viral lytic genes. Overexpression experiments demonstrated that some KSHV circRNAs alter cell proliferation (6).

Given the first roles assigned to some of these viral circRNAs in human herpesviruses, follow-up functional studies should investigate the roles of circRNAs identified in the four loci of the MDV genome. Because the contribution of viral circRNAs in tumorigenesis cannot be evaluated in the human host, natural virus-host models like MDV are needed to assess the role of viral circRNA in lymphomagenesis using deletion mutants.

Without reconsidering the roles of the Meq protein as a functional transcriptional factor controlling a large set of viral and cellular genes, and without questioning data showing the impact of *meq* sequence polymorphisms associated with various levels of MDV virulence (62, 63), the phenotype of *meq* deletion mutants (38, 55, 64–66) might be challenged by the present study. Mechanistic studies on the circRNAs produced from the *meq* transcriptional unit might reveal additional functions or another kind of regulation linked to the major MDV oncogene.

The pp14 locus is poorly characterized until now but has been associated with MDV neurovirulence (50, 56, 57). Interestingly, two features of circular pp14 transcripts can be found in other virus-encoded circular RNAs. First, the origin of MDV DNA replication (OriLyt) is found in the close vicinity of the pp14 transcriptional initiation. Highly expressed circRNAs derived from lytic genes are also encoded from regions surrounding the herpes virus lytic origin of replication in four independent models: EBV (21), MHV68 (22), rLCV (22), KSHV (22). As suggested by Ungerleider et al. (21, 22), this colocalization of OriLyt and circRNA locus raises two nonexclusive assumptions. On the one hand, the initiation of DNA replication may trigger back-splicing events and on the other hand, circular transcripts may act through base pairing at the replication origin to initiate DNA polymerization. The second pp14 feature is that several internal ribosome entry sites (IRES) were previously identified and shown functional, leading to the translation of pp14-b and RLORF9. As described for oncogenic peptides translated from circRNAs in the infection with high-risk human papillomavirus (23), this opens the possibility of pp14-derived proteins translated from MDV circRNAs using these cap-independent initiation sites.

In alphaherpesviruses, LATs belong to a family of RNAs that are spliced from a lncRNA antisense to the mRNA, encoding immediate-early transactivator proteins (39, 45, 46). In MDV, LATs were more thoroughly characterized at their 5′ end, with the identification of several miRNA sequences processed from the first intron (39, 67) and a polymorphism affecting both the LATs promoter and the first exons according to the virulence level of the MDV strain (46, 68, 69). Functional characterization of this region focused on viral miRNAs involved in Marek’s disease pathogenesis (70–72). One of these has been more deeply characterized because it was shown to target two viral genes involved in MDV productive infection and reactivation (39). These functional studies left the 3′ end of the transcript unexplored. In this context, our data pointed out that the 3′ part of the LATs, spanning from exons 8 to 12, is the most productive source of viral circRNAs *in vivo*, paving the way to functional characterization of this part of the LATs.

Regarding ERL, the last genomic locus where circRNAs were detected, a single study presented the hyperediting of a long and highly spliced ncRNA by ADAR-1 (44). Edited circRNA identification from this peculiar transcript fosters better knowledge of the role of these products antisense to pp14, of viral miRNAs and *meq*.

Research on circRNA recently enlightened a new back-splicing pattern (3–7). The footprint left by the splicing machinery did not indicate any known canonical nucleotide sequence, being independent of both U2- and U12-driven splicing processing. In the context of our study, two independent approaches (Fig. 5 to 7) led to the description of noncanonically spliced transcripts. Therefore, we suggest that noncanonical circRNA production might be a hallmark of MDV infection. In recent studies reporting on viral circRNAs, noncanonical back-splice junctions were also observed in KSHV and EBV (21, 73). The precise mechanism behind this alternative splicing process is unknown yet. Because reverse genetics systems offer a straightforward approach for different viruses, the biogenesis of these noncanonical circular spliced transcripts might be tackled in these viral models. ICP27, a conserved protein in herpesviruses, was previously associated with splicing disruption in the context of viral infections (74–76). Making use of the available tools in MDV, ICP27 implication in noncanonical circRNA biogenesis should be addressed by using recombinant viruses deleted for ICP27 (77) or by using overexpression vectors (39, 74). Recent knowledge about splicing may also shed new light on this regulation. Notably, emerging RNA modification studies have described a link between back-splicing and adenosine methylation (reviewed in reference (78)). Further studies should address the involvement of both the methylation enzyme METTL3 as well as ICP27 in circRNA biogenesis in Marek’s disease context.

In conclusion, our study is the first report of viral circRNA expression in MDV infection and MDV-induced lymphoma *in vitro* and tumor samples collected from infected animals. Because viral circRNA expression correlates with transcriptional units of key virulence factors, the associated genes deserve a deeper characterization. The high diversity of back-spliced junctions discovered in our data set opens several perspectives regarding circRNA biogenesis.

## MATERIALS AND METHODS

### Cell lines and viruses.

MSB1 cells are CD4^+^ T lymphocytes isolated from a chicken latently infected by the BC1 strain of MDV. These cells are derived from spleen lymphoma (79). They were cultured in Roswell Park Memorial Institute medium (RPMI 1640, Lonza) supplemented with 10% of fetal bovine serum, 5% of chicken serum (CS), 25 mM HEPES, 1% penicillin (50 units/mL), and streptomycin (50 μg/mL), 1% nonessential amino acids. They were grown at 41°C with 5% of CO_2_. Reactivation was induced by treatment of either 3 mM NaBu or 5 μM 5’azacytidine (Merck) and was verified by the expression of VP5 as previously described (51, 80, 81).

The embryonic stem cell-derived line-1 (ESCDL-1) (82) was cultured in Dulbecco’s modified Eagle medium (DMEM F12 1:1, Lonza), supplemented with 10% fetal bovine serum, 1% penicillin (50 units/mL) and streptomycin (50 μg/mL), 1% nonessential amino acids, and 1% sodium pyruvate. Cells were maintained at 37°C under 5% CO_2_. The ESCDL-1 cells were infected by the GaHV-2 RB-1B strain (EF523390.1).

*Ex vivo* infections of B and T lymphocytes were carried out using the RB-1B strain and following the guidelines described by Schermuly and collaborators (52) with small adaptations of the infection procedure (63, 83). *In vivo* samples used in this study (spleen, feather follicles (FFE), and peripheral blood lymphocytes (PBL)) were obtained from previous experiments (51). They were conducted following Belgian and European laws, notably Directive 2010/63/EU. The ethics committees of Sciensano approved the two sets of experiments that we used in the present study (file numbers being LA1230174 and 20191016-03).

### RNA extraction and circRNA enrichment.

Total RNA was extracted from MDV-infected cells by using TRI Reagent (Invitrogen). Residual DNA was removed with DNase I (NEB) according to the manufacturer’s instructions. After that, to remove linear RNAs, an RNase R (Lucigen) treatment was performed. 4 μg of total RNA was treated with 20 U of RNase R for 20 min at 37°C. Before and after the RNase R treatment, the samples were extracted with phenol-chloroform (Sigma).

RNase R-treated RNA was then reverse-transcribed by using random primers (50 μM) and Superscript III (Invitrogen) reverse transcriptase following the manufacturer’s protocol. The efficiency of the RNase R treatment was evaluated by amplifying one host and one viral circular RNA and their linear counterparts from treated or untreated RNA (Fig. 2).

The resulting cDNAs were amplified by PCR (35 cycles of denaturation [94°C for 30 s], annealing [55 to 60°C for 30 s], and extension [72°C for 1.30 min]) in a final volume of 50 μL containing 1.25 U of GoTaq Polymerase G2 (Promega) and 0.2 mM each primer in the reaction buffer provided by the manufacturer. All the primers used in this study are reported in Table 1. For all the positive PCRs, amplicons were purified by using the NucleoSpin Gel and PCR Clean-up kit (Macherey-Nagel) and inserted into the pGEM-T easy vector (Promega). Around 20 clones were sequenced by Sanger sequencing (Eurofins Genomics) and the corresponding sequences were aligned against the MDV RB-1B genome sequence (Geneious software; Biomatters). To limit the false-positive detection of circRNA production caused by PCR artifacts, clones that did not respect the U2 canonical splice sites (GT/AG) were considered valid only if no more than 5 similar nucleotides were present at both sites of the back-splice junction.

### Bioinformatic analyses.

Two data sets from previous experiments (20, 47) were downloaded from the SRA database (GEO accession numbers: GSE138600 and GSE166240). The first set of data ((20); data available under GSE138600) was obtained from infected chickens with (TA^+^) and without (TA^−^) MDV-induced T CD4^+^ lymphomas and was analyzed through our pipeline. The second set of data ((47); data available under GSE166240) were gathered from chicken embryonic fibroblasts (CEF) infected by the Md5 strain of MDV. All the data obtained from this experiment were analyzed through our pipeline. Note that in the present study, the sequence libraries (GSM5066726, Md5-1 input; GSM5066727, Md5-2 input; GSM5066728, Md5-3 input) correspond to the total RNA extract following circular transcripts enrichment.

The reads were trimmed using Trimmomatic (84) following the publisher’s protocol. The process included the trimming of the adapters, quality trimming (Sliding Window 4:15), and a minimal length filter of 70 nucleotides. The duplicated reads from PCR artifacts were deleted using dedupe.sh from the BBMap package (85). The reads were aligned on the MDV-Md5 genome (NC_002229) for the first time using Burrows-Wheeler Aligner (86) with options set on “mem -a -T15” and the unmapped reads were deleted using Samtools (Genome Research Ltd., 2021) according to the publisher’s instructions.

The reads were filtered with a succession of in-house Python scripts. First, all the spliced reads were filtered by accepting only the reads presenting at least one primary and one supplementary alignment.

Next, the reads presenting a back-splicing signal were extracted. This filter consisted of the analyses of all the different mappings of each read individually. They were sorted following their location. If a sequence proximal in the read was aligned at a distal position in the genomic sequence (and vice versa), it was considered a potential circularization signal, and both the back-spliced junction and donor and acceptor splice sites were stored in a new file with the read IDs. To increase the specificity of our circRNA detection, several filters were put in place deleting (i) the reads that map repetitive sequences; (ii) the reads that present back-splice sites distant by more than 5000 bp on the genome; (iii) the reads with no crossmatch between their back-splice junction and the two corresponding splice sites; (iv) the reads reported with incomplete back-splice junctions or splice sites.

The back-splice junctions were counted according to their similarity. Ninety-five percent of similarity was considered a match allowing thereby one mismatch in the back-splice junction. The same 95% of similarity was also required between the reported back-splice sites. A filter was designed to delete the reads presenting long stretches (more than 5 nucleotides) of similarities at donor and acceptor back-splice sites. The idea was to avoid false positives due to PCR artifacts.

Using the ID list extracted from this count, the complete reads were extracted from the first SAM file to exhibit a proper alignment, processed with Samtools, and drawn using Geneious.

### Data availability.

All data and bioinformatic codes are available on reasonable request to Damien Coupeau (damien.coupeau@unamur.be).
